# Chain-specificity of laminin α1-5 LG45 modules in the recognition of carbohydrate-linked receptors and intramolecular binding

**DOI:** 10.1038/s41598-023-37533-y

**Published:** 2023-06-27

**Authors:** Masumi Matsunuma, Ryuji Kan, Yuji Yamada, Keisuke Hamada, Motoi Kanagawa, Motoyoshi Nomizu, Yamato Kikkawa

**Affiliations:** 1 Department of Clinical Biochemistry, Tokyo University of Pharmacy and Life Sciences, 1432-1 Horinouchi, Hachioji, Tokyo 192-0392 Japan; 2 Department of Cell Biology and Molecular Medicine, Ehime University Graduate School of Medicine, Toon, Ehime 791-0295 Japan

**Keywords:** Cell adhesion, Extracellular matrix

## Abstract

Laminins are a family of heterotrimers composed of α-, β-, and γ-chains in the basement membrane. Five α chains contain laminin globular (LG) domain consisting of five tandem modules (LG1-5 modules) at their C-terminus. Each LG45 modules is connected to a compact cloverleaf-shaped structure of LG1-3 through a flexible linker. Although the accumulated studies of the LG45 modules have suggested differences in each α chain regarding the binding of carbohydrate chain and intramolecular interaction, this remains unclear. In this study, to characterize their functions comparatively, we produced recombinant proteins of LG45 modules of human laminin α1-5 chains. Dystroglycan (DG) modified with matriglycan readily bound to the LG45 modules of α1 and α2 chains but not to the other α chains. In contrast, heparin bound to the LG45 modules of the α chains, except for α2. The binding of heparan sulfate/heparin-linked syndecans (SDCs) to LG45 modules was influenced by their core proteins. Furthermore, the α1 and α4LG45 modules bound to SDCs in a pH-dependent manner. A cell adhesion assay showed that HEK293 cells could readily adhere to the LG45 modules of α3-5 chains through a combination of SDCs and integrins. Moreover, α5LG45 modules bound to the E8 fragment, which includes the C-terminus of the laminin coiled-coil (LCC) domain and LG1-3 modules, but α2LG45 modules did not. The results suggested that although α5LG45 modules was fixed within the LG domain, α2LG45 modules was freely placed in the vicinity of LG1–3. Our findings provide information for investigation of the structural and functional diversity of basement membranes.

## Introduction

**Figure 1:**
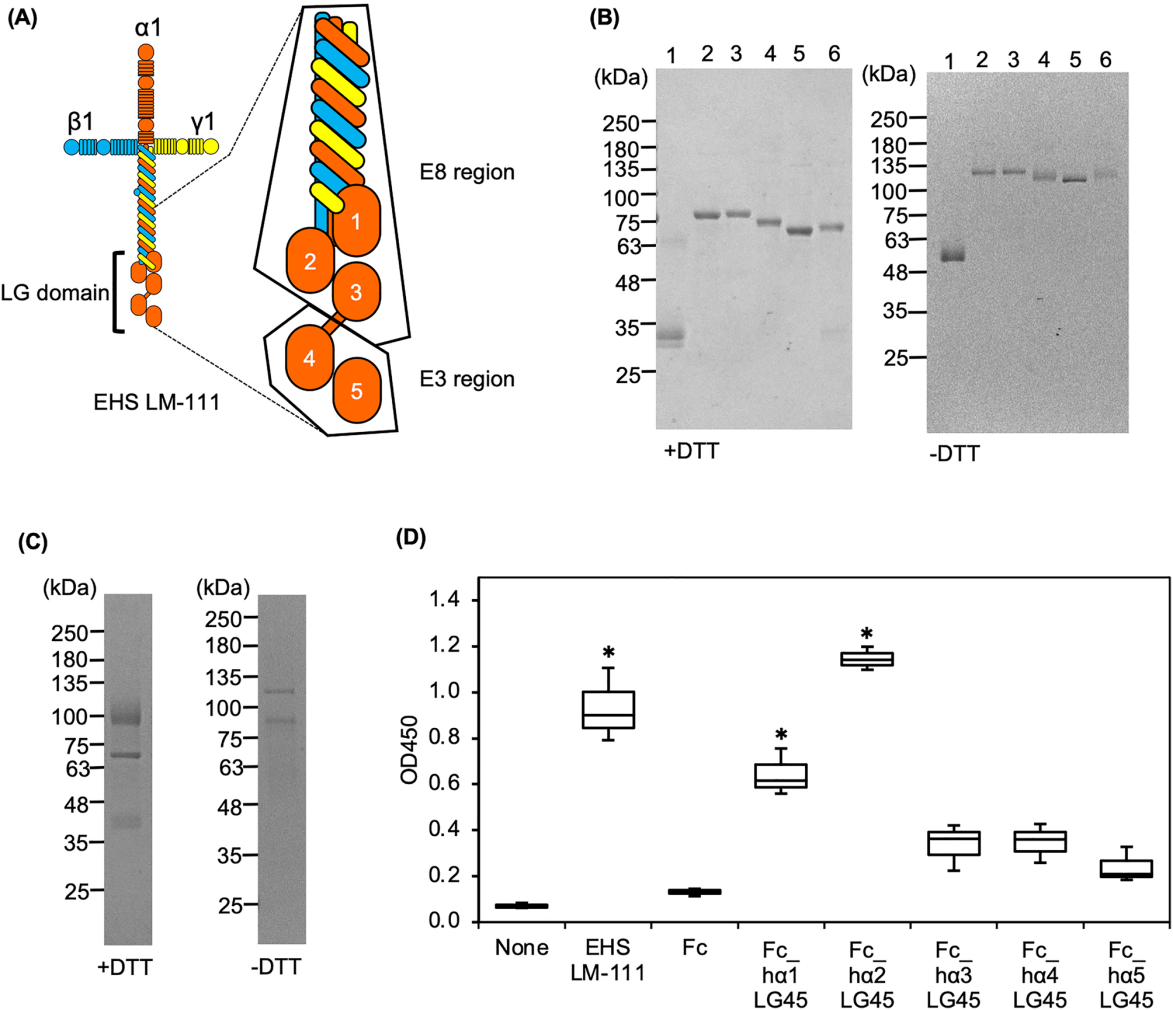
Binding of α-DG to human laminin α1-5 LG45 modules. (**A**) Schematic diagrams of laminin E3 and E8 regions. (**B**) Purification of Fc-tagged laminin α1-5 LG45 modules. Fc (Fc_tag, lane 1), Fc_hα1LG45 (laminin α1 LG45, lane 2), Fc_hα2LG45 (laminin α2 LG45, lane 3), Fc_hα3LG45 (laminin α3 LG45, lane 4), Fc_hα4LG45 (laminin α4 LG45, lane 5) and Fc_hα5LG45 (laminin α5 LG45, lane 6) purified from the conditioned media of transfectants were subjected to SDS-PAGE on a 5–15% gel under reducing (+ DTT) and non-reducing (− DTT) conditions. Original gel images are presented in Supplementary Fig. S1. (**C**) Purification of mouse α-DG fused with Fc-tag (MsDG_Fc). The purified protein was subjected to SDS-PAGE as described above. (**D**) Binding of MsDG_Fc to laminin α1–5 LG45 proteins immobilized to microtiterplate. The bound DG_Fc was detected with anti-α-DG monoclonal antibody and quantified as described in “Methods”. Mouse EHS laminin-111 (EHS LM-111) was used as a positive control. The coating efficiency of recombinant proteins on microtiterplates is presented in Supplementary Fig. S2. Box-and-whisker plots show median, 25th and 75th percentiles, and minimum and maximum values (n = 3 independent experiments). Data were analyzed by one-way ANOVA with Tukey’s multiple-comparison test, *P < 0.01.

Laminins are a large family of multidomain trimeric basement membrane proteins that influence the behaviour of associated cells, such as adhesion, migration, and differentiation^1^. All laminins are composed of three subunits designated as α, β, and γ chains and form the cross- or T-shaped heterotrimers. Five α-, three β-, and three γ-chains have been identified, and 19 different laminin heterotrimeric isoforms are synthesised and secreted by cells^1^. The existence of multiple chains generates not only molecular but also structural and functional diversity in basement membranes. Of the three subunits, the five laminin α chains share a large globular domain in their C-terminal region (LG domain). The receptor-binding sites of laminins are mainly located within the LG domain^2^. The LG domain of the α chain also consists of five homologous modules of ~ 200 residues each (LG1–LG5). These five modules are divided into LG1-3 and LG45, which are involved in the binding of integrin and/or carbohydrate-linked receptors. LG1-3 forms a compact cloverleaf-shaped structure connected to LG45 through a flexible linker^3–5^. The major integrin binding site in laminins requires the native structure of the E8 region, which consists of the C-terminus of the heterotrimeric coiled-coil and the LG1-3 modules of the α chain^6,7^ (Fig. 1A). A recent study has shown that the coiled-coil is attached perpendicularly to a triangular arrangement of LG1-3 modules of the α chain and that the γ1 tail lies between LG1 and LG2^5^. Integrins α3β1, α6β1, α6β4, and α7β1 serve as laminin receptors that bind to the E8 region^8^. Laminin–integrin interactions are required not only for morphogenesis and tissue function but also for the long-term self-renewal of human stem cells in the laboratory^9,10^. The LG45 modules is also termed as the E3 region^6^. Although integrin α2β1 bound to the E3 region of α1 chain^11^, the binding of integrins on the LG45 modules of the other α chains remains a controversial issue.

The LG domain is present in several other extracellular matrix proteins, including agrin, perlecan, neurexin-1, pikachurin, slit, and EYS^12^. The LG domains also bind carbohydrate ligands such as heparan sulfate/heparin, sulfated glycolipids, and matriglycan polysaccharides. The LG45 modules of the laminin LG domains are a major binding region for the carbohydrate chain. Dystroglycan (DG) consists of a heavily glycosylated extracellular α subunit and a membrane-spanning β subunit. Glycosaminoglycan-like polysaccharide, termed matriglycan, of α-DG binds to the LG45 modules of laminins α1, α2, and α5 in Ca^2+^-dependent manner^13^. Heparan sulfate/heparin is a carbohydrate present in proteoglycans such as perlecan, agrin, and syndecans (SDCs). Several studies have shown that heparan sulfate/heparin binds to LG45 modules of laminin α chains^14^. In contrast to matriglycan, heparan sulfate/heparin binding does not require divalent cation^12^. The heparin binding sites of LG45 modules are also served as reservoirs of growth factors such as VEGF and PDGF^15^. Therefore, the characterization of LG45 modules is required for the induction of various differentiations from stem cells cultured on laminins.

Crystallographic studies revealed that the LG1-3 modules of α1 and α5 chains exhibits a triangular arrangement^3,5^. Although the structures of LG1-3 and LG45 modules in the other α chains have not been investigated, they should be similar. The LG45 modules is further connected to LG1-3 through a flexible linker. However, it is unclear how the LG45 modules is placed in the vicinity of LG1–3LG45 modules.

In this study, we comparatively characterized the functions of αLG45 modules. We first produced the recombinant proteins comprising LG45 modules of human laminin α1-5 chains. The purified recombinant proteins were then evaluated for binding to dystroglycan and heparin/SDCs. We also examined the cell adhesion activity of LG45 modules and their intramolecular binding. Our findings revealed the individual characteristic features of the recombinant proteins for each activity. Each α chain LG 45 modules should provide functional diversity to basement membranes.

## Results

### α-DG binding property of laminin α LG45 modules

DG is a transmembrane receptor consisting of α and β subunits that forms a tight link between the basement membrane and the cytoskeleton^13^. The mannosyl-*O*–linked carbohydrate of the α subunit is involved in binding to LG domains of extracellular matrices, such as laminin, agrin, and perlecan^12^. Accumulated evidences have indicated that laminin α LG45 modules are involved in laminin binding to α-DG. However, because the LG45 modules of each α chain have been studied individually, comparative studies are inadequate. To re-evaluate the binding of α-DG, we prepared recombinant human laminin α1-5 LG45 modules fused with human IgG_1_ Fc from the conditioned media. Human IgG_1_ Fc was used as a control protein. SDS-PAGE analysis of the purified protein revealed a single protein band corresponding to the expected size (Fig. 1B). The self-aggregating insoluble forms were not observed in the fractions stored at 4 °C. We also produced recombinant mouse α-DG fused to human IgG_1_ Fc (MsDG_Fc). SDS-PAGE analysis exhibited two bands under reducing and non-reducing conditions (Fig. 1C). Furin processes N-terminal region of α-DG and creates the lower molecule binding to laminin-111^16^. The lower band seemed to be a cleaved form of α-DG. A solid-phase binding assay of MsDG_Fc was performed using recombinant LG45 modules immobilized to microtiter plates. The series of recombinant proteins were equally coated on the microtiter plates (Supplementary Fig. S2). Bound MsDG_Fc was detected using an anti-α-DG rat monoclonal antibody. The α2LG45 module was tightly bound to α-DG (Fig. 1D). Although the α1LG45 module had significant affinity for α-DG, other recombinant proteins exhibited low binding affinity at this coating concentration. The results indicate that the α-DG binding property of laminin α LG45 modules could be classified into two groups.

### Heparin binding of laminin α LG45 modules

**Figure 2:**
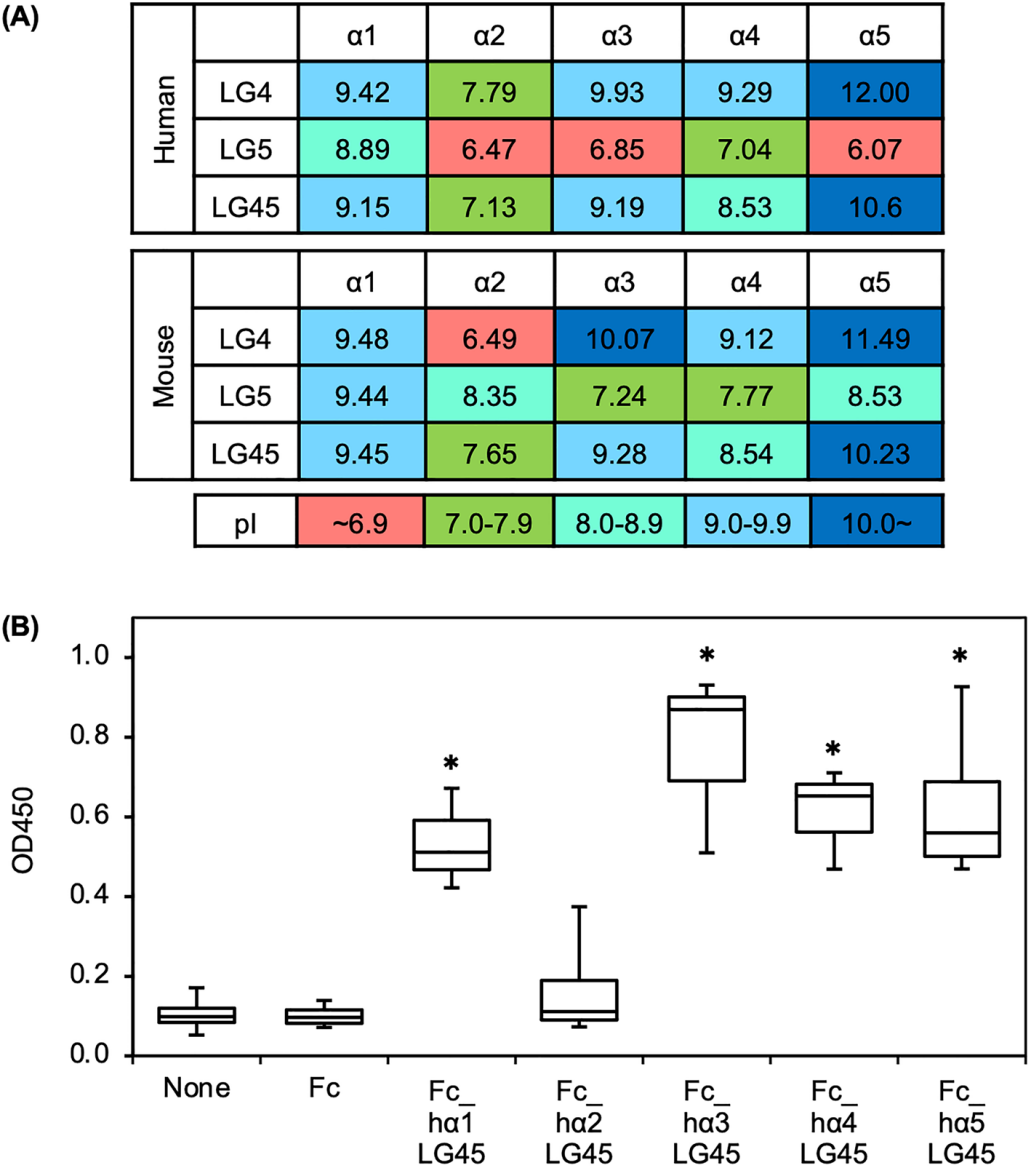
Heparin binding to human laminin α1-5 LG45 modules. (**A**) pIs of human and mouse laminin α LG45 modules. The pIs were calculated at Protein Identification and Analysis Tools on the Expasy Server (https://web.expasy.org/protparam/). (**B**) Heparin binding of laminin α1–5 LG45 modules immobilized to microtiterplate. The bound heparin was detected with horseradish peroxidase-conjugated streptavidin. Box-and-whisker plots show median, 25th and 75th percentiles, and minimum and maximum values (n = 3 independent experiments). Data were analyzed by one-way ANOVA with Tukey’s multiple-comparison test, *P < 0.05.

Heparin, which is a highly sulfated form of heparan sulfate, is involved in cell–matrix and matrix–matrix interactions^14^. Previous studies have indicated that the LG45 modules is involved in heparin binding activity on laminins^2,14^. As described above, comparative studies are inadequate. Heparin acts as a cation exchanger owing to its polyanionic nature, and it can bind to cationic proteins. Because the charge of the LG45 modules is dependent on their isoelectric points, we calculated these based on their amino acid sequences (Fig. 2A). The pI of α2LG45 was exclusively neutral in humans and mice. The pI of the other LG45 modules was basic, indicating that they are positively charged under neutral conditions. The calculation on each LG module also suggested that the LG4 module influenced the pI of the LG45 modules rather than the LG5 module. In this study, a heparin-binding assay was performed under neutral conditions (pH = 7.5). As expected, the LG45 modules of the α chains, except for α2, were significantly bound to heparin (Fig. 2B).

### SDC binding to laminin α LG45 modules

**Figure 3:**
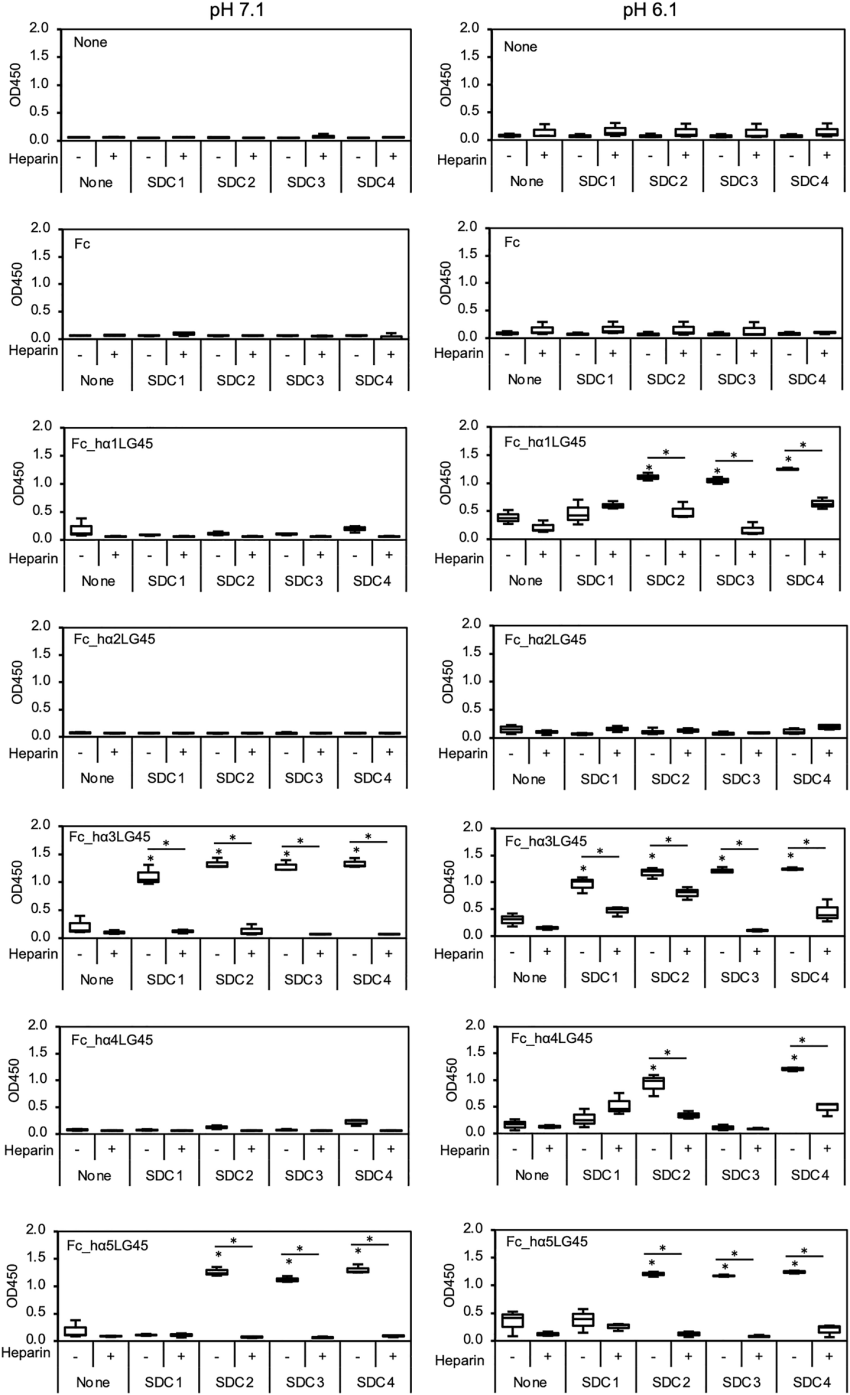
Solid-phase binding assays of recombinant proteins to immobilized SDCs. 96-well microtiter plates were coated with 10 μg/mL of extracellular domains of SDC1, 2, 3, and 4. After blocking, the wells were incubated with 10 μg/mL of the recombinant LG45 modules. The recombinant Fc protein was used as control. The binding assays were performed in 150 mM NaCl with 10 mM BisTris-HCl at pH = 6.1 (right) or pH = 7.1 (left), without or with 10 μg/mL of heparin, as indicated. The bound recombinant proteins were detected with anti-human IgG_1_ Fc antibody. Box-and-whisker plots show median, 25th and 75th percentiles, and minimum and maximum values (n = 3 from 3 independent experiments). Data were analyzed by one-way ANOVA with Tukey’s multiple-comparison test, *P < 0.01.

SDCs are a small family of heparan sulfate proteoglycans that are type I transmembrane glycoproteins^17^. The heparin-binding activities of the LG45 modules led us to examine whether they interact with SDCs, which are composed of heparan sulfate and core proteins. We first performed solid-phase binding assays of the LG45 modules to immobilised SDCs under neutral conditions (pH = 7.1) (Fig. 3). The LG45 modules of the α3 and α5 chains were bound to SDC2, 3, and 4. The binding of SDC1 was exclusively observed in the α3LG45 modules. The LG45 modules of the other α chains did not bind to SDCs. Because the pIs of LG45 modules in α chains, except for α2, are basic, they are positively charged under acidic conditions and bind more effectively to heparan sulfate. Solid-phase binding assays were performed under acidic conditions (pH = 6.1). The LG45 modules of the α1 and α4 chains bound to syndecans in a pH-dependent manner. The binding of SDC1 was not observed in the LG45 modules of other α chains. Moreover, the α4LG45 module did not bind to SDC3 under acidic conditions. The binding of the α3 and α5LG45 modules to SDCs was observed in the same way as in the neutral condition. The SDC bindings to the LG45 modules of α3 and α5 chains under neutral conditions were completely inhibited in the presence of heparin. On the other hand, the inhibitory effects of heparin under acidic conditions were incomplete in SDC bindings to the α1, α3, and α4LG45 modules.

### Characterization of cell adhesion to laminin α LG45 modules

**Figure 4:**
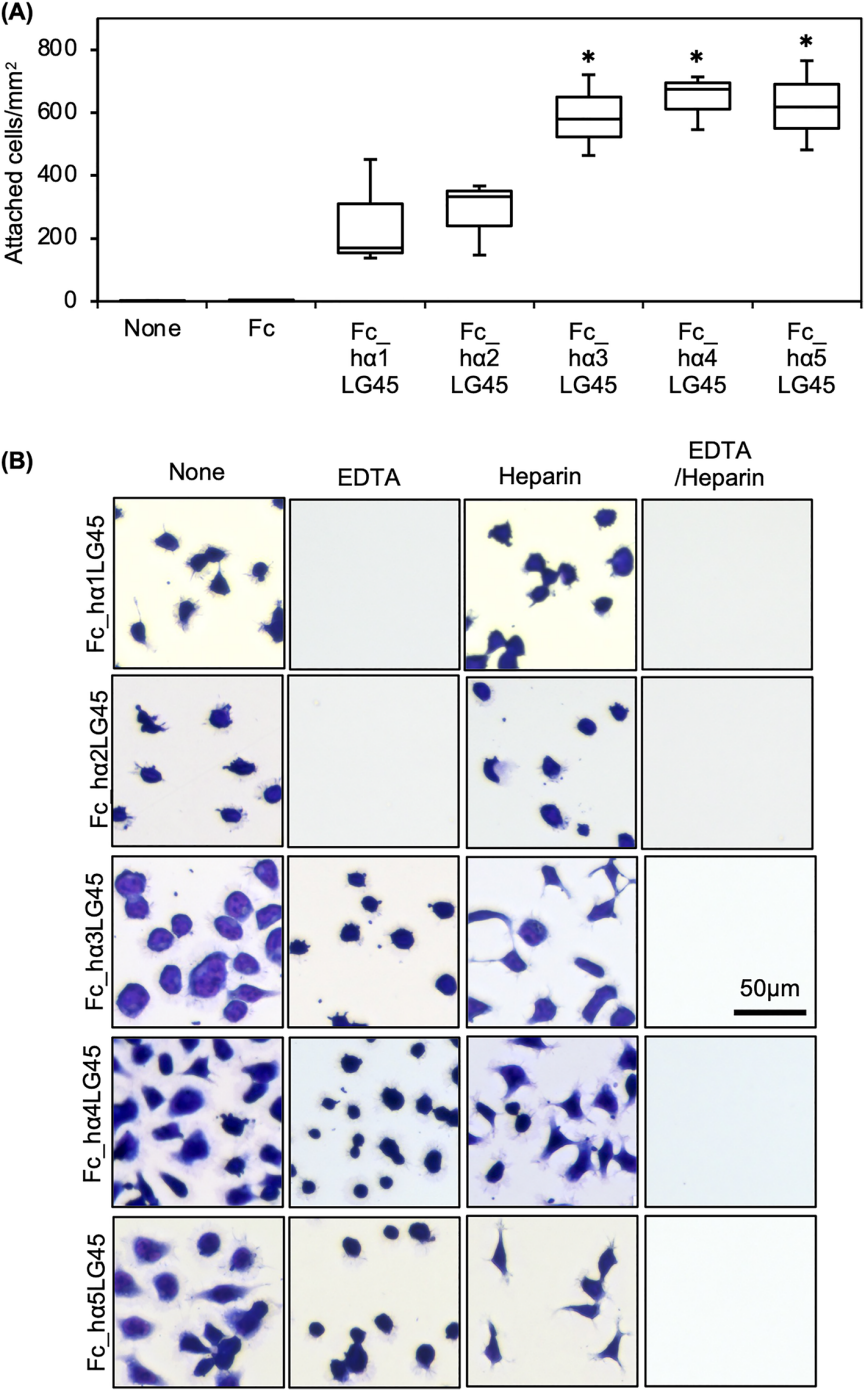
Cell adhesion to laminin α1-5 LG45 modules. (**A**) Cell adhesion assay using HEK293 cells. Attached cells were stained and counted under a microscope. Box-and-whisker plots show median, 25th and 75th percentiles, and minimum and maximum values (n = 3 independent experiments). Data were analyzed by one-way ANOVA with Tukey’s multiple-comparison test, *P < 0.01. (**B**) Influences of EDTA or/and heparin on adhesion of HEK293 cells to the LG45 modules. HEK293 cells pre-incubated with EDTA or/and heparin were added to the recombinant protein-coated wells. The attached cells were observed as described above. Quantification of cell attachment was presented in Supplementary Fig. S3.

The DG and SDC binding of the LG45 modules led us to hypothesise that they modulate cell adhesion to laminins. Therefore, we explored the cell adhesion activity of the LG45 modules (Fig. 4A). HEK293 cells suspended in serum-free DMEM were plated in wells coated with recombinant proteins. The cells readily adhered to the LG45 modules of α3–5 chains and spread on the substrata. Although the α1 and α2LG45 modules exhibited cell adhesion activities, they were lower than those of the α3–5 chains. To characterise cell adhesion to the LG45 modules, cell morphology and attachment were observed in the presence of EDTA or/and heparin (Fig. 4B and Supplementary Fig. S3). Although EDTA completely inhibited cell adhesion to the LG45 modules of the α1 and α2 chains, heparin did not influence the cell morphology of the substrata. EDTA also impaired cell spreading on the LG45 modules of α3–5 chains. Heparin promoted the elongation of pseudopodia in the substrata. Moreover, the combination of EDTA and heparin completely inhibited cell adhesion to the LG45 modules of α3–5 chains.

**Figure 5:**
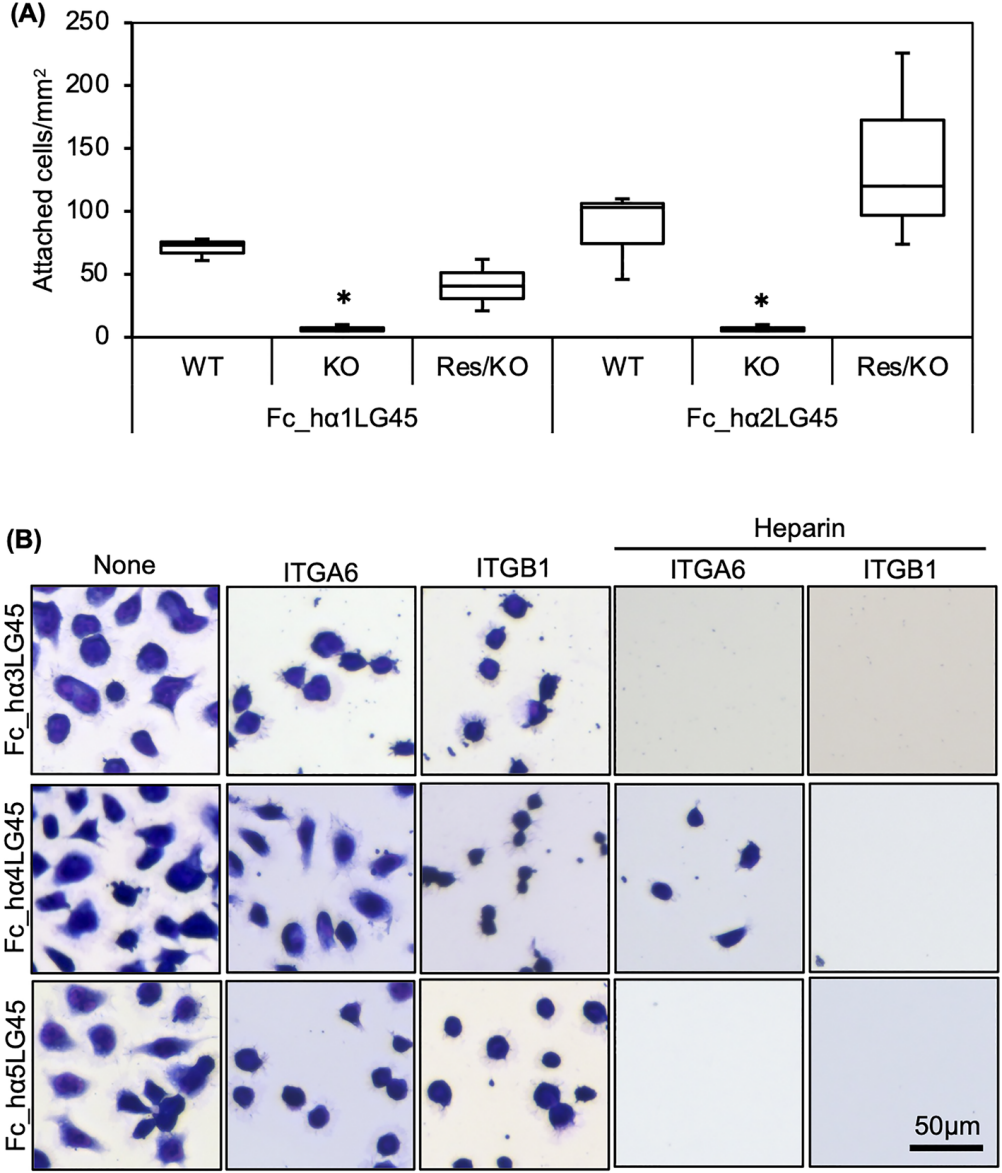
Identification of cell surface receptors binding to human laminin α1-5 LG45 modules. (**A**) Adhesion of HEK293 (WT), *FKTN*-knockout HEK293 (KO), and *FKTN*-rescued KO cells (Res/KO) to laminin α1 and α2 LG45 modules. The KO cells are lacking the glycosylation on α-DG associated with laminin binding. The attached cells were stained and counted under a microscope. (**B**) Inhibitory effects of antibodies to integrin α6 (ITGA6) and β1 (ITGB1) on cell adhesion in the presence of heparin. HEK293 cells pre-incubated with antibodies and heparin were added to the recombinant protein-coated wells. The attached cells were observed by microscopy.

The complete inhibition of cell adhesion to the LG45 modules of α1 and α2 chains by EDTA suggests that this is mediated by DG. To confirm the involvement of DG, we performed a cell adhesion assay using *FKTN*-deficient 293 cells that lacked glycosylation associated with laminin binding (Supplementary Fig. S4A). As expected, the deficient cells did not adhere to α1 or α2LG45 modules (Fig. 5A). To exclude the off-target effects of CRISPR/Cas9 editing, the KO cells overexpressing the *FKTN* gene were used. The rescued cells restored the adhesion to α1 and α2LG45 modules. In addition to SDC2 and 3, HEK239 cells expressed integrin α1-6β1 and αVβ1 (Supplementary Fig. S4B). Furthermore, to identify integrin binding to α3-5LG45 modules, cell morphology was observed in the presence of function-blocking antibodies to integrins (Fig. 5B and Supplementary Fig. S5). The results showed that although anti-integrin α6 and β1 antibodies did not inhibit cell adhesion, they did impair cell spreading. Furthermore, both antibodies inhibited cell adhesion to α3 and α5LG45 modules in the presence of heparin, indicating that SDCs and integrin α6β1 simultaneously serve as receptors. The combination of heparin and anti-integrin α6 antibody incompletely inhibited cell adhesion to the α4LG45 modules, suggesting involvement of integrin β1 paired with another α subunit.

### The binding of LG45 modules to laminins

**Figure 6:**
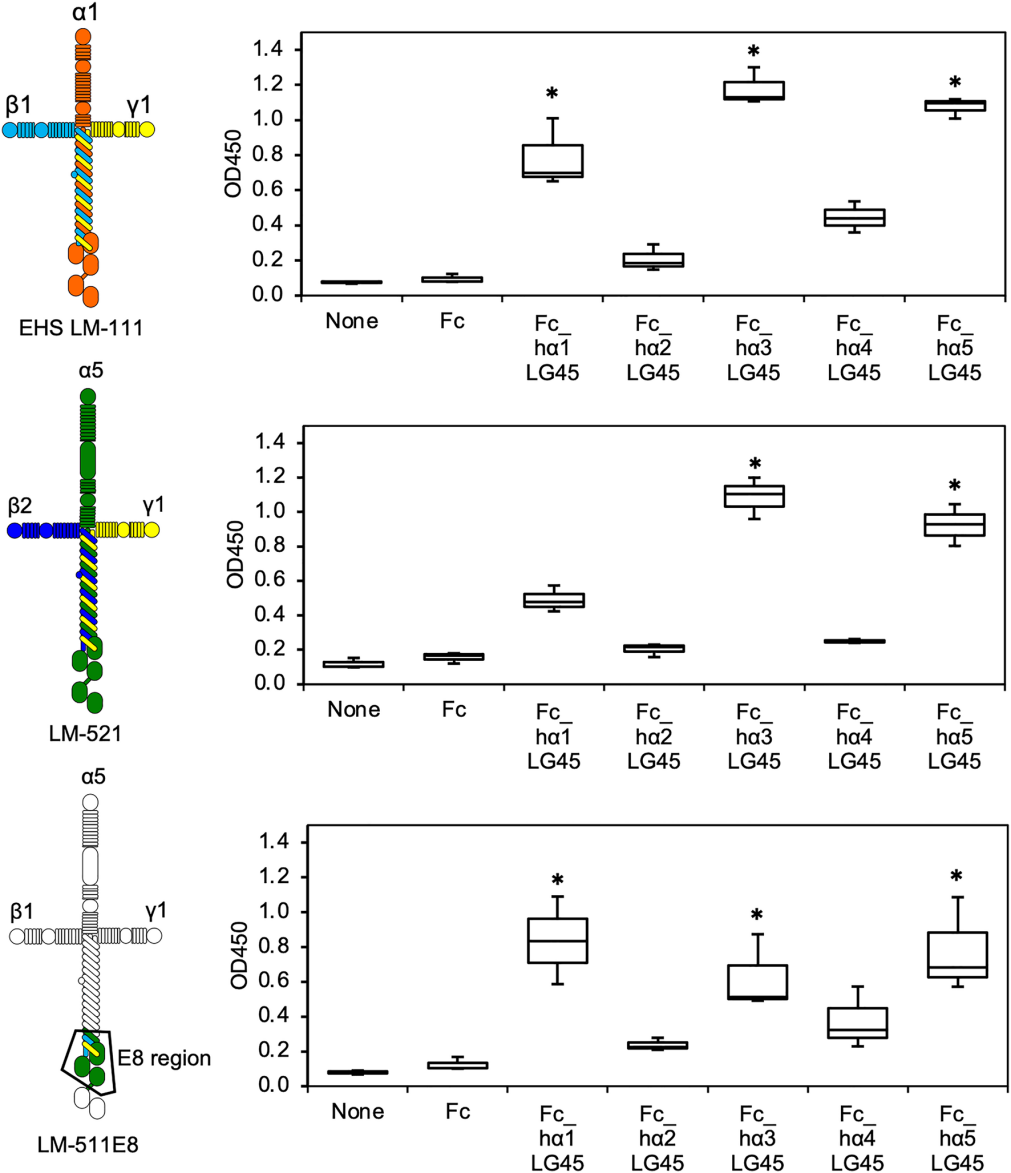
Solid-phase binding assays of laminin LG45 modules to the immobilized laminins. 96-well microtiter plates were coated with mouse EHS laminin-111 (EHS LM-111), laminin-521 (LM-521), and laminin-511-E8 (LM-511E8). The bound recombinant proteins were detected with anti-human IgG_1_ Fc antibody. Box-and-whisker plots show median, 25th and 75th percentiles, and minimum and maximum values (n = 3 independent experiments). Data were analyzed by one-way ANOVA with Tukey’s multiple-comparison test, *P < 0.01.

Each LG4 module is linked to LG3 through linker regions composed of 39–66 amino acid residues. Since it was unclear how the LG45 modules are placed in whole laminin containing the LG domain, we examined their binding to laminins. The solid-phase binding assay showed that the LG45 modules of the α1, α3 and α5 chains bound to EHS laminin-111 consisting of α1, β1, and γ1 (Fig. 6). To investigate the binding, we further examined the binding of LG45 modules to laminin-521, which consists of α5, β2, and γ1. The results indicated that although the α3 and α5 LG45 modules bound to laminin-521, the binding of α1LG45 modules was decreased. To narrow down the binding region, we performed a solid-phase binding assay using the E8 fragments of laminin-511 (α5, β1, γ1) and -221 (α2, β2, γ1). The results showed that the binding site of the α3 and α5 LG45 modules was localized in the E8 region (Fig. 6 and Supplementary Fig. S6). The binding of α1LG45 modules was also restored in the E8 fragment containing the laminin β1 C-terminus, indicating that the β chain influenced the binding of α1LG45 modules.

### Characterization of the binding of LG45 modules to the E8 fragment

**Figure 7:**
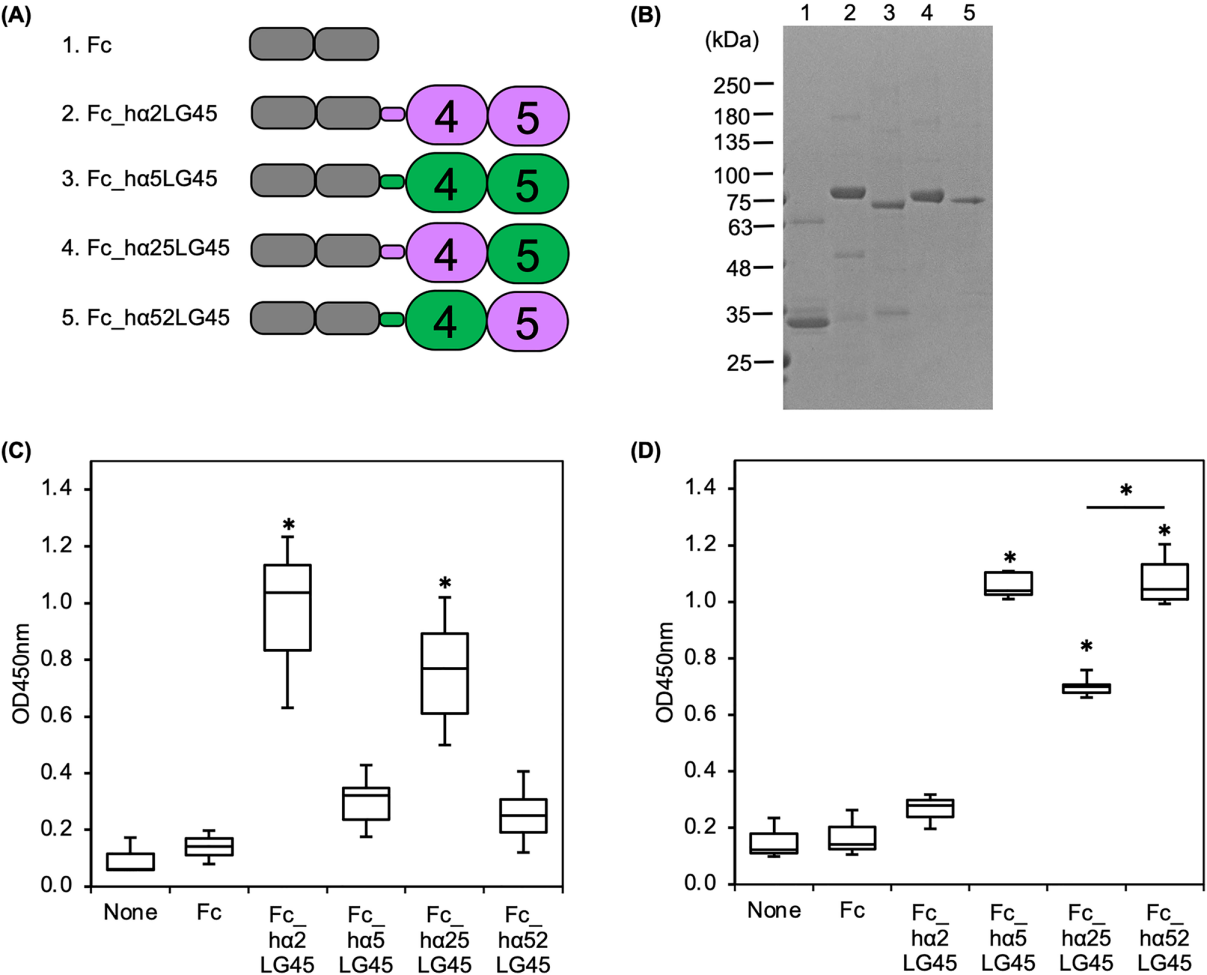
Intramolecular binding of laminin LG45 modules. (**A**) Schematic diagrams of chimeric recombinant proteins. (**B**) SDS-PAGE of the purified recombinant proteins. Fc (Fc_tag, lane 1), Fc_hα2LG45 (laminin α2 LG45, lane 2), Fc_hα5LG45 (laminin α5 LG45, lane 3), Fc_hα25LG45 (laminin α2LG4 and α5LG5, lane 4), and Fc_hα52LG45 (laminin α5LG4 and α2LG5, lane 5). Original gel is presented in Supplementary Fig. S1. (**C**) Binding of MsDG_Fc to chimeric LG45 proteins. The bound MsDG_Fc was detected with anti-α-DG monoclonal antibody and quantified as described in Methods. Box-and-whisker plots show median, 25th and 75th percentiles, and minimum and maximum values (n = 3 independent experiments). Data were analyzed by one-way ANOVA with Tukey’s multiple-comparison test, *P < 0.01. (**D**) Solid-phase binding assays of chimeric LG45 proteins to the immobilized laminin-221-E8. The bound recombinant proteins were detected with anti-human IgG_1_ Fc antibody. Data were statistically analyzed as described above. *P < 0.05.

To narrow down the E8 fragment-binding site on the α5LG45 modules, we produced chimeric recombinant proteins (Fig. 7A). Each module of α5LG45 was replaced by a corresponding α2LG module that did not bind to the E8 fragment. The chimeric recombinant proteins were purified from the conditioned media and verified by SDS-PAGE (Fig. 7B). Because the chimeric proteins exhibited the expected DG binding, their structures were ascertained (Fig. 7C). We performed a solid-phase binding assay of chimeric LG45 modules to the immobilised the E8 fragment of laminin-221 (Fig. 7D). Both chimeric recombinant proteins were bound to the E8 fragment, indicating that the binding region was across the entire α5LG45.

## Discussion

The structure of the LG45 modules of α1 and α2 chains has been well studied and provides predictions for the other α chains^12^. As suggested by the structural studies, laminin α1-5 LG45 modules exhibited functional diversity (Table 1). The α2LG45 module is a major region for α-DG binding and is associated with tight linkages in the sarcolemma^18^. As shown in previous studies^19^, our recombinant α2LG45 modules tightly bound to α-DG. Furthermore, although α1LG45 modules readily bound to α-DG, binding of α3-5LG45 modules was not significantly observed at this concentration (10 μg/mL). α1 and α2LG45 coordinate calcium ions and bind to α-DG in a Ca^2+^-dependent manner^12^. Amino acid sequence comparison predicted that laminin α3, α4, and α5 chains do not have calcium-binding sites analogous to those of α1 and α2LG45^14^. Ca^2+^ ion coordination requires potent binding of matriglycan polysaccharide^12^. Previous studies have reported that although α-DG binds LG4 modules of the laminin α4 and α5 chains, both bindings have less affinity than α1 and α2 chains^20,21^. The weak binding of α-DG appears to be due to the lack of calcium binding sites and indicates the possibility that α-DG can bind weakly to laminin α LG45 modules in a Ca^2+^-independent manner. DG binding also depends on molecular weight of matriglycan synthesiyzed by LARGE^13,22^. The high molecular weight-matriglycan modified in LARGE-overexpressing cells may be able to bind to LG45 modules of the laminin α4 and α5 chains in a Ca^2+^-independent manner. Although the binding of α3LG45 modules to α-DG has not been reported yet, it should be similar to the α4 and α5 chains. Our findings indicate that the α-DG binding properties of laminin α LG45 modules can be classified into two groups.

**Table 1:** Summary of the binding properties of αLG45 modules.

	α-dystroglycan	Heparin	Syndecans								Cell adhesion of HEK293	Laminin binding
			SDC1		SDC2		SDC3		SDC4			
			pH6.1	pH7.1	pH6.1	pH7.1	pH6.1	pH7.1	pH6.1	pH7.1		
hα1LG45	+++	+++	−	−	+++	−	+++	−	+++	−	+ (DG)	++
hα2LG45	+++	−	−	−	−	−	−	−	−	−	+ (DG)	−
hα3LG45	+/−	+++	++	+++	+++	+++	+++	+++	+++	+++	++ (SDC s, integrin α6β1)	+++
hα4LG45	+/−	+++	−	−	+	−	−	−	+++	−	++ (SDCs, integrin α?β1)	±
hα5LG45	+/−	+++	−	−	+++	+++	+++	+++	+++	+++	++ (SDCs, integrin α6β1)	+++

Our results showed that heparin can bind to recombinant LG45 modules of human laminin α chains, except for α2. Previous studies have reported that the recombinant protein comprising mouse laminin α2LG45 modules exhibits heparin-binding activity^23,24^. To address this controversial issue, we compared the amino acid sequences of laminin α2 LG45 modules in humans and mice. We found that although the pI of the α2LG5 module is basic in mice, it is acidic in humans (Fig. 2A). Talts et al. also reported that heparin binds to the mouse α2LG5 module but not LG4^23^. The positive charge of the mouse α2LG5 module under neutral conditions appears to be involved in heparin binding. Dystroglycanopathies are caused by the aberrant glycosylation of α-DG^13^. The dystroglycanopathy model mice are less severe than patients^25^. The difference in symptoms and onset between dystroglycanopathy patients and model mice may be due to distinct heparin binding to α2LG45 modules.

SDCs frequently possess heparan sulfate glycosaminoglycan chains^26^. Therefore, the straightforward interpretation of the heparin binding property was that all SDCs bound to recombinant LG45 modules of human laminin α chains, except for α2. However, different SDC bindings were observed in the recombinant proteins. Because all SDCs bind to the α3LG45 modules, their heparan sulfate glycosaminoglycan chains seem to be enough to function in the binding. Our results also showed that inhibitory effects of heparin in SDCs binding under acidic condition are incomplete. Although we need to do more experiments are required, the core proteins should impact the binding of heparan sulfate glycosaminoglycan chains. The α3LG45 modules exclusively binds to SDC1. Carulli et al. reported that α3LG45 modules bind to heparan sulfate glycosaminoglycan chains of SDC1, but not its core protein^27^. The core protein of SDC1 should influence the α chain-specific binding of heparan sulfate glycosaminoglycan chains. We also showed that acidic conditions can bind SDCs to LG45 modules of α1 and α4 chains. However, the α4LG45 module did not bind to SDC3. In addition to heparan sulfate glycosaminoglycan, chondroitin sulfate is also present on SDC1 and 3^28^. The α4LG45 module may not be able to bind to SDCs modified with chondroitin sulfate.

Our results indicate that the LG45 modules of α3, α4, and α5 chains are recognised by integrin α6β1. Furthermore, α4 LG45 modules bind to additional β1-containing integrins. However, because the integrin binding of LG45 modules is much weaker than that of LG1-3 modules, its influence on cell adhesion to whole laminin should be negligible. As described in previous studies^2^, laminin α LG45 modules mainly serve as ligands for non-integrins, such as DG and SDCs. DG is broadly expressed not only in skeletal muscle but also in various tissues^29^. However, the role of DG in various cells, other than myofibres, remains unclear. Because the recombinant human laminin α2LG45 modules are exclusively recognised by α-DG, it would be useful for analysing receptor functions in non-muscle cells.

The bindings of laminin-511 E8 fragment (α5, β1, γ1) to the α3 and α5 LG45 modules are comparable to those of laminin-221 E8 fragment (α2, β2, γ1). Although there still remains a possibility that α and β chains in the E8 region are involved in the bindings of the α3 and α5 LG45 modules, C-terminus of γ1 chain shared in the E8 fragments seems to play a pivotal role in the binding. Takizawa et al. reported that laminin-511 binds to integrin α6β1 via the bottom side of LG1–3^5^. Furthermore, the laminin γ1 tail lies between LG1 and LG2, and Glu^1607^ is required for integrin α6β1 binding. Because integrin α6β1 binds to whole laminin-511 conatining LG45 modules, it is unlikely that α5LG45 binds to the bottom side containing laminin γ1 tail. Structured study further showed that γ1 chain in the terminus of LCC domain faces to the inside of ladle-shaped laminin-511 E8 fragment^5^. Therefore, we hypothesised that α5LG45 modules binds to the terminus of LCC domain containing γ1 chain and are located on the upper side of LG1–3. Our results also show that the α3LG45 modules bind to laminin-332 (Supplementary Fig. S6). Although the α3LG45 module is enzymatically cleaved from laminin-332^30^, the fragment appears to be trapped in the cleaved laminin. The recombinant α2 and α4LG45 modules did not bind to laminins, suggesting that α2 and α4LG45 modules are freely placed in the vicinity of LG1–3. The flexibility of the α2LG45 modules may be important for binding to α-DG. Laminin β2 decreases the binding of α1LG45 modules but not to those of α3 and α5LG45 modules. In laminin-121, consisting of α1, β2, and γ1, the freely placed α1LG45 modules may easily access α-DG. Our results also show that the E8 fragment-binding is across the entire α5LG45. However, because Fc_hα25LG45, lacking the α5LG4 module, significantly reduces the binding affinity, the module primarily appeares to bind to the E8 fragment.

Genetic analysis has revealed that mutations in human laminin α chain LG45 modules are pathogenic^31^. In our recent study, a *LAMA5* variant carrying p.Val3687Met was identified in a family with hereditary focal segmental glomerulosclerosis^32^. The mutation was localized in the LG45 modules of the α5 chain. Similarly, mutations in the LG45 modules of α2 and α3 cause muscular dystrophy congenital type 1A^33^ and junctional epidermolysis bullosa^34^, respectively. Our findings will be useful for clarifying the pathogenic molecular basis of LG45 modules.

## Methods

### Antibodies and reagents

Monoclonal antibodies against human integrin α2 (P1E6), α3 (P1B5), α4 (P4G9), α5 (P1D6), α6 (P5G10), αV (P3G8), and β1 (AIIB2) were purified using Protein G Sepharose^®^ (Cytiva, Marlborough, MA, USA) from conditioned media of hybridoma cells purchased from the Developmental Studies Hybridoma Bank (Iowa City, IA). Anti-integrin α1 (FB12) and anti-α-DG (3D7 and IIH6) antibodies were purchased from Merck (Kenilworth, NJ, USA). Anti-SDC1 (MI15) and 4 (5G9) antibodies were purchased from BioLegend (San Diego, CA, USA) and Santa Cruz Biotechnology (Dallas, TX, USA), respectively. Anti-SDC2 (305515) and 3 (374412) antibodies were purchased from R&D Systems (Minneapolis, MN, USA). Human recombinant laminin-521 (LM-521) and laminin-332 (LM-332) were purchased from BioLamina (Sundbyberg, Sweden) and ReproCell (Yokohama, Japan), respectively. iMatrix-511 (LM-511E8) and -221 (LM-221E8) cells were purchased from Nippi (Tokyo, Japan). Mouse EHS laminin-111 (EHS LM-111) was obtained from BD Biosciences (San Jose, CA, USA) and from Dr. Takako Sasaki (Oita University School of Medicine, Oita, Japan). Recombinant SDC1, 2, 3, and 4 were purchased from R&D Systems. Biotinylated heparin with an average mass of 12.5 kDa was obtained from Celsus Laboratories, Inc. (Cincinnati, OH, USA).

### Cell culture

Human embryonic kidney (HEK293) cells were purchased from the American Type Culture Collection (Manassas, VA) and maintained in DMEM containing 10% fetal calf serum. *FKTN*-knockout HEK293 (KO), and *FKTN*-rescued KO (Res/KO) cells were prepared as described in our previous study^35^.

### Construction of expression vectors

cDNA clones encoding full-length human laminin α1 and α5 were purchased from Kazusa Genome Technologies (Chiba, Japan). DNA fragments encoding the LG45 modules of human laminin α2–4 chains were also synthesised by Thermo Fisher Scientific (Waltham, MA, USA) (Supplementary Table S1). They were used to construct expression vectors. Because LG45 modules are located at the C-terminus of the laminin α chain, the signal sequence, Fc-tag, and linker were fused at the N-terminus of the fragments. The modified MO90 vector^36^ was used as a template for the amplification of the DNA fragment encoding the human laminin γ2 signal sequence, human IgG_1_ Fc, and ASTGS linker. The amplified fragment was digested with MfeI and XbaI and inserted into the EcoRI and XbaI sites of pcDNA3.1Zeo (+). Fragments encoding LG45 modules of human laminin α1–5 chains were amplified using the primer sets described in Supplementary Table S2. The PCR products were seamlessly joined to DNA encoding the ASTGS linker using In-Fusion^®^ Snap Assembly Master Mix (Takara Bio Inc., Shiga, Japan).

### Expression and purification of recombinant proteins

HEK293 cells transfected with expression vectors were selected using 100 μg/mL Zeocin (Thermo Fisher Scientific). All transfectants were grown to confluence in culture dishes with DMEM containing 10% FBS and antibiotics. Confluent cells were cultured in serum-free DMEM for four days. Conditioned media were prepared by sequential centrifugation at 1000 rpm for 5 min and at 10,000 rpm for 10 min. Recombinant proteins were purified from the culture media using Protein A Sepharose^®^ (Cytiva). The eluted fractions were dialysed against PBS(−). The purified proteins were separated by SDS-PAGE using 5–20% gels under reducing and non-reducing conditions. The separated proteins were stained with Coomassie Brilliant Blue. Expression vectors of cDNAs encoding mouse α-DG fused with human IgG_1_ Fc (MsDG_Fc) and control Fc were constructed as described in our previous studies^37,38^. Recombinant proteins were produced using the Expi293™ Expression System according to the manufacturer's instructions. Recombinant proteins were purified as described above.

### Solid-phase binding assays

For the α-DG binding assay, 96-well microtiter plates were coated with 10 μg/mL of Fc-tagged recombinant proteins and 200 μg/mL of mouse EHS laminin-111 (EHS LM-111). After blocking with PBS(−) containing 1% BSA, 10 μg/mL of α-DG recombinant protein in 10 mM Tris–HCl pH 7.5, 150 mM NaCl, 1 mM CaCl_2_, and 1 mM MgCl_2_ containing 0.05% Tween 20 was incubated at 4 °C overnight. Bound MsDG_Fc was detected using an anti-α-DG monoclonal antibody. After incubation for 60 min at room temperature, the bound antibody was detected by adding anti-rat IgG-conjugated horseradish peroxidase, followed by the addition of 1 mg/mL *o*-phenylenediamine and 0.012% H_2_O_2_. The absorbance was measured at 450 nm using a Multiskan™ GO microplate spectrophotometre (Thermo Fisher Scientific).

Heparin-binding assays were carried out with recombinant proteins coated onto high-protein-binding capacity 96-well microtiter plates (AGC Techno Glass, Shizuoka, Japan). The plates were blocked with PBS(−) containing 1% BSA for 60 min at room temperature. Biotinylated heparin (10 μg/mL) in 10 mM Tris–HCl (pH 7.5) containing 150 mM NaCl was added to each well. After incubation for 60 min at room temperature, bound heparin was detected by the addition of streptavidin-conjugated horseradish peroxidase and quantified as described above.

For SDC binding assay, 96-well microtiter plates were coated with 10 μg/mL of SDC1, 2, 3 and 4 (R&D systems). After blocking with PBS(−) containing 1% BSA, 10 μg/mL of Fc-tagged recombinant proteins in 10 mM BisTris-HCl (pH 7.1 or pH6.1), 150 mM NaCl, containing 0.05% Tween 20 were incubated at 4 °C overnight. The bound recombinant proteins were detected with a biotinylated anti-human IgG_1_ Fc antibody (Jackson ImmunoResearch) and quantified as described above.

For the laminin-binding assay, 96-well microtiter plates were coated with 10 μg/mL of EHS laminin-111, laminin-521, laminin-511-E8, laminin-221-E8, and 3 μg/mL laminin-332. After blocking with PBS(−) containing 1% BSA, 5 μg/mL of Fc-tagged recombinant proteins in 10 mM Tris–HCl (pH 7.5), 150 mM NaCl, and 0.05% Tween 20 was incubated at 4 °C overnight. The bound recombinant proteins were detected and quantified as described above.

### Cell attachment assays

For adhesion assays, 96-well microtiter plates (AGC Techno Glass) were coated with recombinant proteins and blocked with PBS(−) containing 1% BSA. Dissociated cells were suspended in serum-free DMEM and plated at 4 × 10^5^ cells/50 μL/well. After incubation at 37 °C for 30 min, the attached cells were stained using Diff-Quik (Sysmex, Kobe, Japan). The stained cells were counted using an image cytometer BZ-X800 (Keyence, Osaka, Japan). To identify the receptors for laminin, 10 μg/mL of monoclonal antibodies against different integrins were pre-incubated individually with cells in 50 μL of serum-free medium (4 × 10^5^ cells/well) at room temperature for 10 min.

### Flow cytometric analysis

Cells were detached with cell dissociation buffer (Thermo Fisher Scientific) and suspended in PBS(−) containing 0.1% BSA and 1 mM EDTA. The suspended cells were incubated with antibodies described above. Following washing with PBS(−), cells were incubated with Alexa 488-labeled secondary antibody. The cells were then analyzed on a FACSCelesta flow cytometer (Becton Dickinson, San Jose, CA).

### Statistical analysis

Comparisons between groups were performed using one-way ANOVA with Tukey’s multiple-comparison test. Statistical analyses were performed using R Studio v.1.4.1717. Significance is depicted as follows: *P < 0.05, **P < 0.01.

## Supplementary Information

Supplementary Information.

## Data Availability

All data generated or analysed during this study are included in this published article and its supplementary information files.
