# Supplementary material for: Chain-specificity of laminin α1-5 LG45 modules in the recognition of carbohydrate-linked receptors and intramolecular binding
Source: Sci Rep. 2023 Jun 27;13:10430. doi: 10.1038/s41598-023-37533-y (PMC10300086; doi:10.1038/s41598-023-37533-y)
Supplement: Supplementary file 1 — Supplementary Information. [file 41598_2023_37533_MOESM1_ESM.pdf]

Supplementary information

Chain-specificity of laminin α1-5 LG45 modules in the recognition of carbohydrate-linked receptors and intramolecular binding

Masumi Matsunuma[1], Ryuji Kan[1], Yuji Yamada[1], Keisuke Hamada[1], Motoi Kanagawa[2], Motoyoshi Nomizu[1], Yamato Kikkawa[1*]

[1] Department of Clinical Biochemistry, Tokyo University of Pharmacy and Life Sciences, Tokyo 192-0392, Japan

[2] Department of Cell Biology and Molecular Medicine, Ehime University Graduate School of Medicine, Toon, Ehime 791-0295, Japan

*Corresponding author

Table S1. Synthetic DNA	
_hα2LG45_	gcttctactggatccgaattcGTTCTGACACATGGTCCTTGTGCTGCAGAATCAGAACCAGCTCTTTT<br>GATAGGGAGCAAGCAGTTCGGGCTTTCAAGAAACAGTCACATTGCAATAGCATTTGAT<br>GACACCAAAGTTAAAAACCGTCTCACAATAGAGTTGGAAGTAAGAACCGAAGCTGAAT<br>CCGGCTTGCTTTTTACATGGCTCGCATCAATCATGCTGATTTTGCAACAGTTCAGCTGA<br>GAAATGGATTGCCCTACTTCAGCTATGACTTGGGGAGTGGGGACACCCACACCATGATC<br>CCCACCAAAATCAATGATGGCCAGTGGCACAAGATTAAGATAATGAGAAGTAAGCAAG<br>AAGGAATACTTTATGTAGATGGGGCTTCCAACAGAACCATCAGTCCCAAAAAAGCCGA<br>CATCCTGGATGTCGTGGGAATGCTGTATGTTGGTGGGTTACCCATCAACTACACTACCC<br>GAAGAATTGGTCCAGTGACCTATAGCATTGATGGCTGCGTCAGGAATCTCCACATGGCA<br>GAGGCCCCTGCCGATCTGGAACAACCCACCTCCAGCTTCCATGTTGGGACATGTTTTGC<br>AAATGCTCAGAGGGGAACATATTTTGACGGAACCGGTTTTGCCAAAGCAGTTGGTGGAT<br>TCAAAGTGGGATTGGACCTTCTTGTAGAATTTGAATTTCGCACAACTACAACGACTGGA<br>GTTCTTCTGGGGATCAGTAGTCAAAAAATGGATGGAATGGGTATTGAAATGATTGATGA<br>AAAGTTGATGTTTCATGTGGACAATGGTGCGGGCAGATTCACTGCTGTCTATGATGCTG<br>GGGTTCCAGGGCATTTGTGTGATGGACAATGGCATAAAGTCACTGCCAACAAGATCAA<br>ACACCGCATTGAGCTCACAGTCGATGGGAACCAGGTGGAAGCCCAAAGCCCAAACCCA<br>GCATCTACATCAGCTGACACAAATGACCCTGTGTTTGTTGGAGGCTTCCCAGATGACCT<br>CAAGCAGTTTGGCCTAACAACCAGTATTCCGTTCCGAGGTTGCATCAGATCCCTGAAGC<br>TCACCAAAGGCACAGGCAAGCCACTGGAGGTTAATTTTGCCAAGGCCCTGGAACTGAG<br>GGGCGTTCAACCTGTATCATGCCCAGCCAACTAAtctagagggcccgtttaa
_hα3LG45_	gcttctactggatccgaattcAAGGTGTGGCAAGATGCTTGCTCACCACTTCCCAAGACCCAGGCCA<br>ATCATGGAGCCCTCCAGTTTGGGGACATTCCCACCAGCCACTTGCTATTCAAGCTTCCTC<br>AGGAGCTGCTGAAACCCAGGTCACAGTTTGCTGTGGACATGCAGACAACATCCTCCAG<br>AGGACTGGTGTTTCACACGGGCACTAAGAACTCCTTTATGGCTCTTTATCTTTCAAAAG<br>GACGTCTGGTCTTTGCACTGGGGACAGATGGGAAAAAATTGAGGATCAAAAGCAAGGA<br>GAAATGCAATGATGGGAAATGGCACACGGTGGTGTTTGGCCATGATGGGGAAAAGGGG<br>CGCTTGGTTGTGGATGGACTGAGGGCCCGGGAGGGAAGTTTGCCTGGAAACTCCACCAT<br>CAGCATCAGAGCGCCAGTTTACCTGGGATCACCTCCATCAGGGAAACCAAAGAGCCTC<br>CCCACAAACAGCTTTGTGGGATGCCTGAAGAACTTTCAGCTGGATTCAAAACCCTTGTA<br>TACCCCTTCTTCAAGCTTCGGGGTGTCTTCCTGCTTGGGTGGTCCTTTGGAGAAAGGCAT<br>TTATTTCTCTGAAGAAGGAGGTCATGTCGTCTTGGCTCACTCTGTATTGTTGGGGCCAGA<br>ATTTAAGCTTGTTTTCAGCATCCGCCCAAGAAGTCTCACTGGGATCCTAATACACATCG<br>GAAGTCAGCCCGGGAAGCACTTATGTGTTTACCTGGAGGCAGGAAAGGTCACGGCCTC<br>TATGGACAGTGGGGCAGGTGGGACCTCAACGTCGGTCACACCAAAGCAGTCTCTGTGT<br>GATGGACAGTGGCACTCGGTGGCAGTCACCATAAAACAACACATCCTGCACCTGGAAC<br>TGGACACAGACAGTAGCTACACAGCTGGACAGATCCCCTTCCCACCTGCCAGCACTCAA<br>GAGCCACTACACCTTGGAGGTGCTCCAGCCAATTTGACGACACTGAGGATCCCTGTGTG<br>GAAATCATTCTTTGGCTGTCTGAGGAATATTCATGTCAATCACATCCCTGTCCCTGTCAC<br>TGAAGCCTTGGAAGTCCAGGGGCCTGTCAGTCTGAATGGTTGTCCTGACCAGTAAtctagag<br>ggcccgtttaa
_hα4LG45_	gcttctactggatccgaattcACTCCAAGAAACTCTCATTGCCACCTTTCCAACAGCCCTAGAGCAAT<br>AGAGCACGCCTATCAATATGGAGGAACAGCCAACAGCCGCCAAGAGTTTGAACACTTA<br>AAAGGAGATTTTGGTGCCAAATCTCAGTTTTCCATTCGTCTGAGAACTCGTTCCTCCCAT<br>GGCATGATCTTCTATGTCTCAGATCAAGAAGAGAATGACTTCATGACTCTATTTTTGGCC<br>CATGGCCGCTTGGTTTACATGTTTAATGTTGGTCACAAAAAACTGAAGATTAGAAGCCA<br>GGAGAAATACAATGATGGCCTGTGGCATGATGTGATATTTATTCGAGAAAGGAGCAGT<br>GGCCGACTGGTAATTGATGGTCTCCGAGTCCTAGAAGAAAGTCTTCCTCCTACTGAAGC<br>TACCTGGAAAATCAAGGGTCCCATTTATTTGGGAGGTGTGGCTCCTGGAAAGGCTGTGA<br>AAAATGTTCAGATTAACTCCATCTACAGTTTTAGTGGCTGTCTCAGCAATCTCCAGCTCA<br>ATGGGGCCTCCATCACCTCTGCTTCTCAGACATTCAGTGTGACCCCTTGCTTTGAAGGCC<br>CCATGGAAACAGGAACTTACTTTTCAACAGAAGGAGGATACGTGGTTCTtGATGAATCT<br>TTCAATATTGGATTGAAGTTTGAAATTGCATTTGAAGTCCGTCCCAGAAGCAGTTCCGG<br>AACCCTGGTCCACGGCCACAGTGTCAATGGGGAGTACCTAAATGTTCACATGAAAAAT<br>GGACAGGTCATAGTGAAAGTCAATAATGGCATCAGAGATTTTTCCACCTCAGTAACACC<br>CAAGCAGAGTCTCTGTGATGGCAGATGGCACAGAATTACAGTTATTAGAGATTCTAATG<br>TGGTTCAGTTGGATGTGGACTCTGAAGTGAACCATGTGGTTGGACCCCTGAATCCAAAA<br>CCAATaGATCACAGGGAGCCTGTGTTTGTTGGAGGTGTTCCAGAATCTCTACTGACACC<br>ACGCTTGGCCCCCAGCAAACCCTTCACAGGCTGCATACGCCACTTTGTGATTGATGGAC<br>ACCCAGTGAGCTTCAGTAAAGCAGCCCTGGTCAGCGGCGCCGTAAGCATCAACTCCTGT<br>CCAGCAGCCTAAtctagagggcccgtttaa

Table S2. Primer sets		
Protein	Primer	Sequence (5'-3')
sigFc_C	HG2SIG_03	CGCAATTGACCGCCATGCCTGCGCTCTGGCTGGGC
	Fc_06	GCATTCTAGAGGGAATTCGGATCCAGTAGAAGCTTTACCCGGAG ACAGGGAGAGGCT
hα1LG45	HA1G_01.1	GCTTCTACTGGATCCGAATTCCGGGCTTTTCCAGAACAGTGTGTG
	HA1G_02	TTAAACGGGCCCTCTAGATCAGGACTCGGTCCCAGGACAGGA
hα2LG45	HA2G_01	GCTTCTACTGGATCCGAATTCGTTCTGACA
	HA2G_02	TTAAACGGGCCCTCTAGATTAGTTGGCTGG
hα3LG45	ASTGS_EI_01	GCTTCTACTGGATCCGAATTC
	STOP_XaP_02	GGTTTAAACGGGCCCTCTAGATTA
hα4LG45	ASTGS_EI_01	GCTTCTACTGGATCCGAATTC
	STOP_XaP_02	GGTTTAAACGGGCCCTCTAGATTA
hα5LG45	HA5G_01.1	GCTTCTACTGGATCCGAATTCCCCGCCCGGCATCCTGCCTGCATG
	HA5G_02	CTTGCTTCTCGGTCACCTGCAACTGCAAGTAGGGGGGCG
	HA5G_03	TGACCGAGAAGCAAGTCCTGCTGCGGGCGGATGACGGA
	HA5G_04	TTAAACGGGCCCTCTAGACTAGGCGGCTGGGCAGCCACTGGC
hα23LG45	HA2G_01	GCTTCTACTGGATCCGAATTCGTTCTGACA
	HA2G_06	ACCACCCAAACATGTCCCAACATGGAAGCTGGA
	HA3G_07	GGGACATGTTTGGGTGGTCCTTTGGAGAAAGGC
	HA3G_06	CCTGACCAGTAATCTAGAGGGCCCGTTTAA
hα32LG45	HA3G_05	GCTTCTACTGGATCCGAATTCAAGGTGTGG
	HA3G_08	ATTTGCAAAGCAGGAAGACACCCCGAAGCTTGA
	HA2G_05	TCTTCCTGCTTTGCAAATGCTCAGAGGGGAACA
	HA2G_02	TTAAACGGGCCCTCTAGATTAGTTGGCTGG
hα25LG45	HA2G_01	GCTTCTACTGGATCCGAATTCGTTCTGACA
	HA2G_06	GCCCAAGATACATGTCCCAACATGGAAGCTGGA
	HA5G_03	GGGACATGTATCTTGGGCCCCCTGGAGGCGGGC
	HA5G_04	TTAAACGGGCCCTCTAGACTAGGCGGCTGGGCAGCCACTGGC
hα52LG45	HA5G_01.1	GCTTCTACTGGATCCGAATTCCCCGCCCGGCATCCTGCCTGCATG
	HA5G_08	ATTTGCAAAGCAGGGTGTGACCCCTGCCATCCG
	HA2G_05	ACACCCTGCTTTGCAAATGCTCAGAGGGGAACA
	HA2G_02	TTAAACGGGCCCTCTAGATTAGTTGGCTGG

Unedited gel for Figure 1B left panel

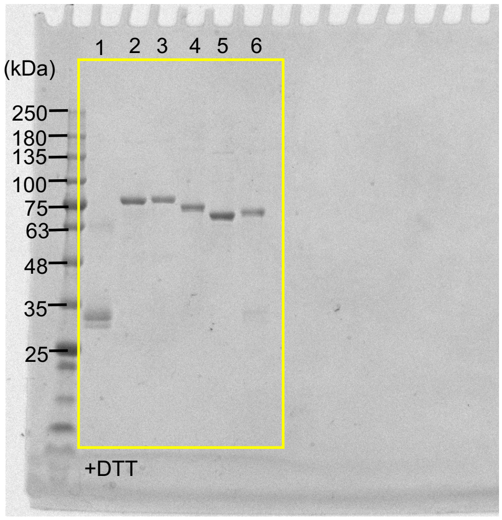

Unedited gel for Figure 1B right panel

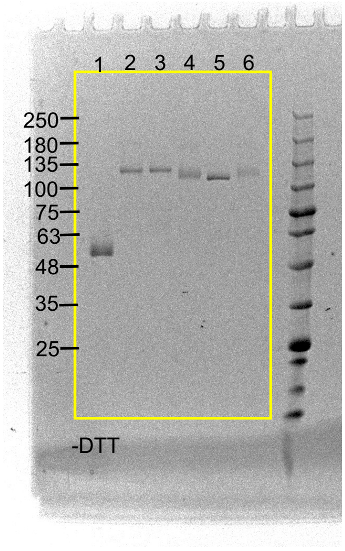

Unedited gel for Figure 1C left panel

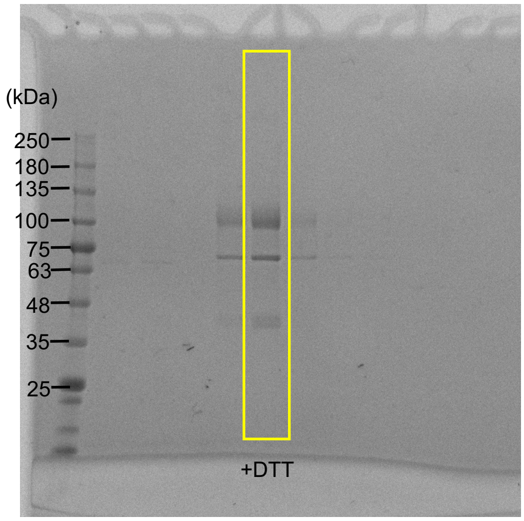

Unedited gel for Figure 1C right panel

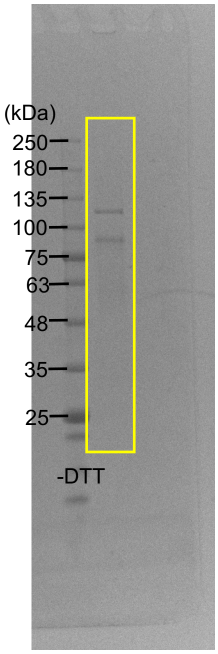

Unedited gel for Figure 7B

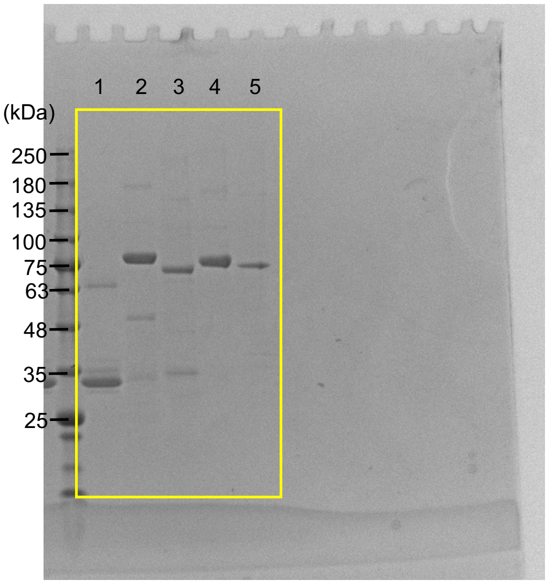

Figure S1. Original gel images

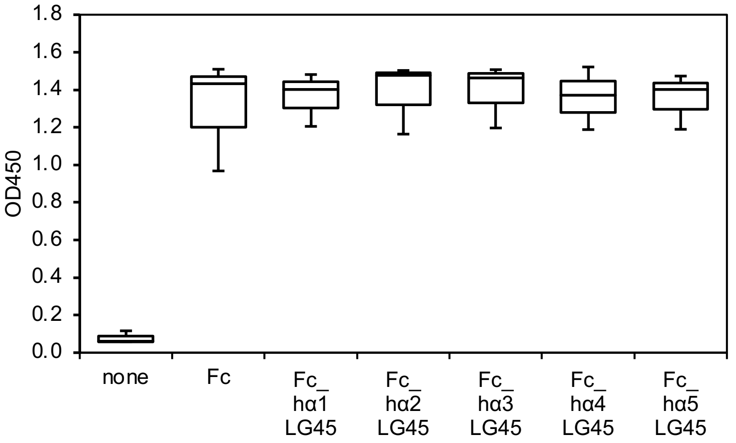

Figure S2. The coating efficiency of α1-5 LG45 modules fused with Fc-tag on a microtiter plate. The coated recombinant proteins were detected with a biotinylated anti-human IgG_1 Fc antibody. The bound antibodies were detected by addition of streptavidin-conjugated horseradish-peroxidase, followed by addition of 1 mg/mL *o*-phenylenediamine and 0.012% H_2O_2. The absorbance was measured at 450 nm with Multiskan™ GO microplate Spectrophotometer (Thermo Fisher Scientific).

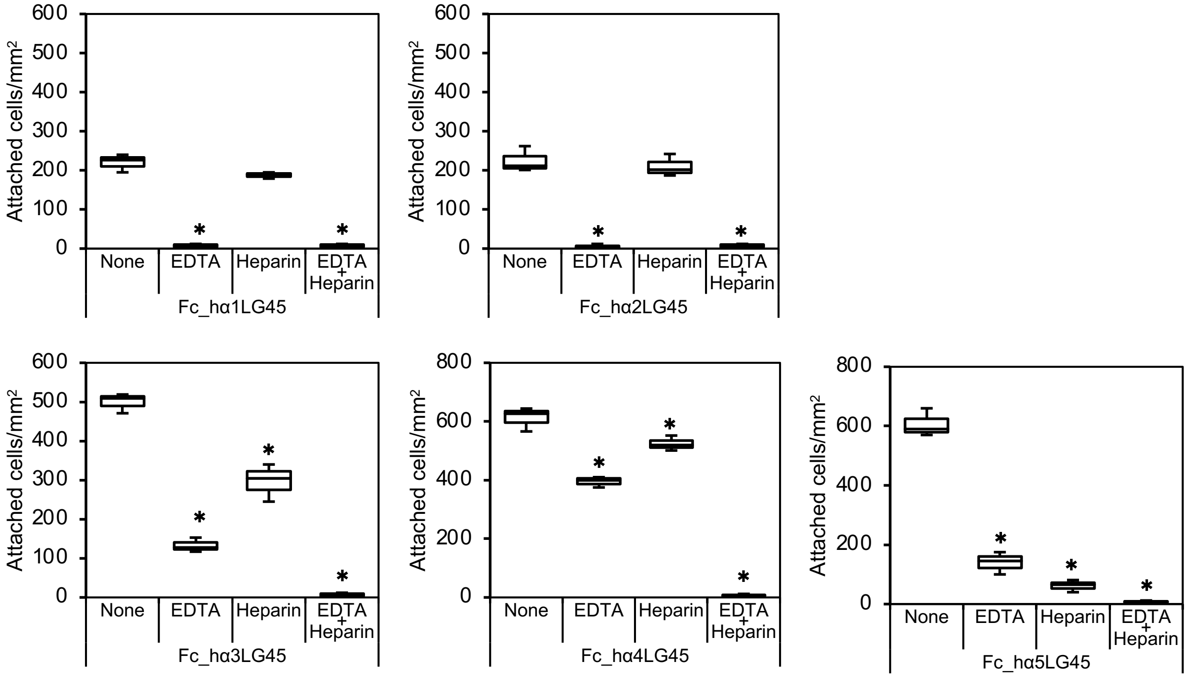

Figure S3. Quantification of cell attachment in the presence of EDTA or/and heparin. HEK293 cells pre-incubated with EDTA or/and heparin were added to the recombinant protein-coated wells. After incubation for 30 min, the attached cells were stained and quantified as described in METHODS. *$P < 0.01$

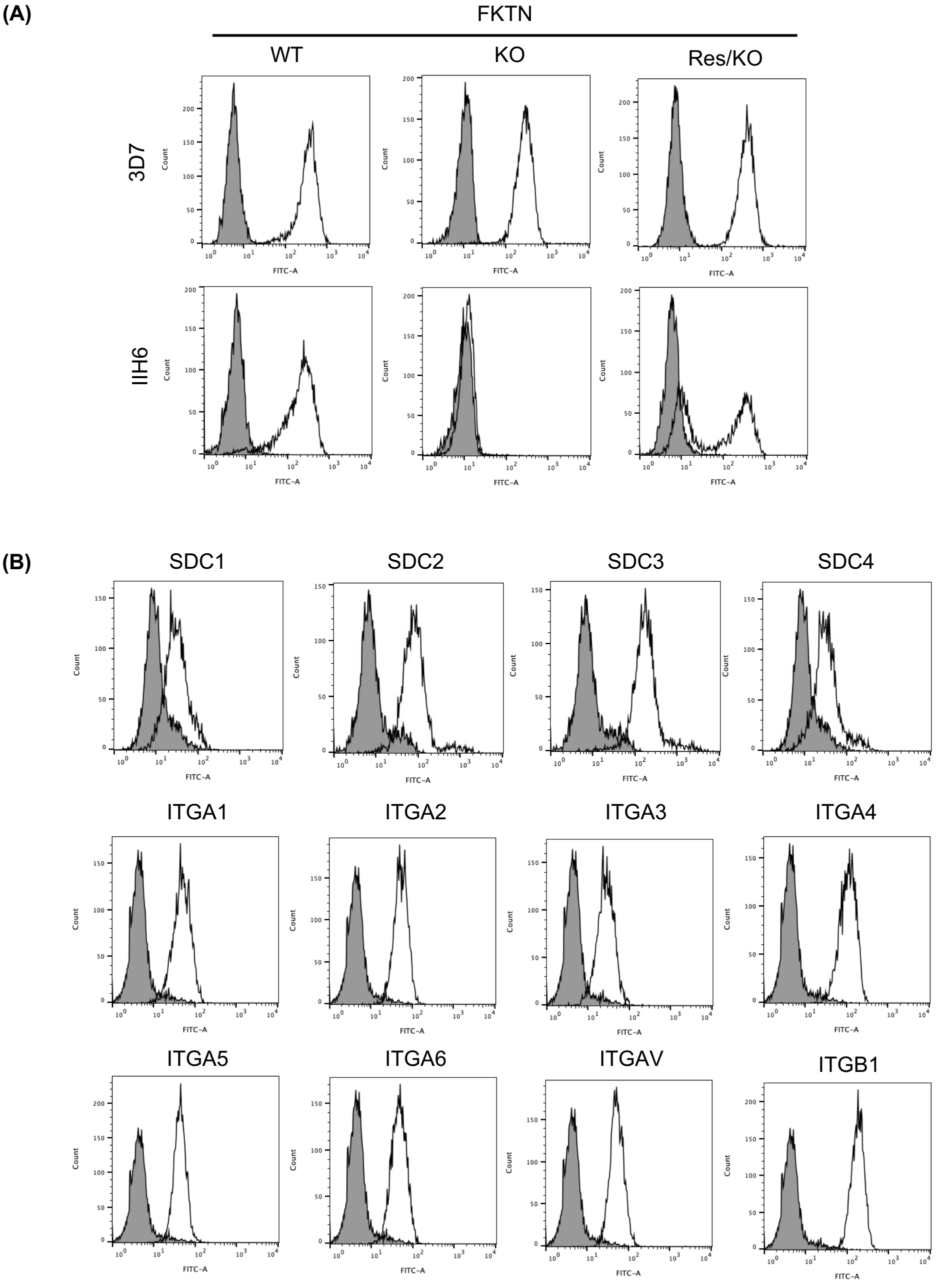

Figure S4. Flow cytometric analyses of DG, syndecans, and integrins expression. (A) Expression of functional α-DG on HEK293 (WT), *FKTN*-knockout HEK293 (KO), and *FKTN*-rescued KO cells (Res/KO). The cells were incubated with two primary antibodies. 3D7 and IIH6 monoclonal antibodies recognize core protein of α-DG and the mannosyl-*o*-linked carbohydrate associated with laminin binding, respectively. The bound antibody is shown as a solid line. The grey area indicates the negative control. (B) Expression of syndecans and integrins on HEK293. The cells were incubated with primary antibodies against syndecan-1 (SDC1), -2 (SDC2), -3 (SDC3), -4 (SDC4), integrin α1 (ITGA1), α2 (ITGA2), α3 (ITGA3), α4 (ITGA4), α5 (ITGA5), α6 (ITGA6), αv (ITGAV), or β1 (ITGB1).

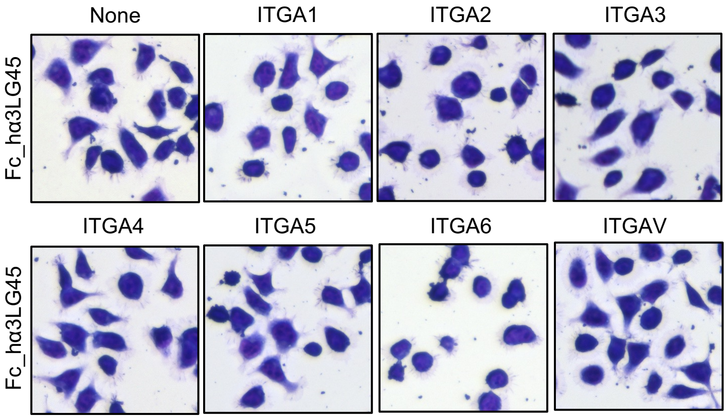

Figure S5. Identification of cell surface receptors binding to laminin α3 LG45 modules. HEK293 cells pre-incubated with antibodies to integrin α1 (ITGA1), α2 (ITGA2), α3 (ITGA3), α4 (ITGA4), α5 (ITGA5), α6 (ITGA6), or αv (ITGAV) were added to the Fc_hα3LG45-coated wells. After incubation for 30 min, the attached cells were stained and observed by microscopy.

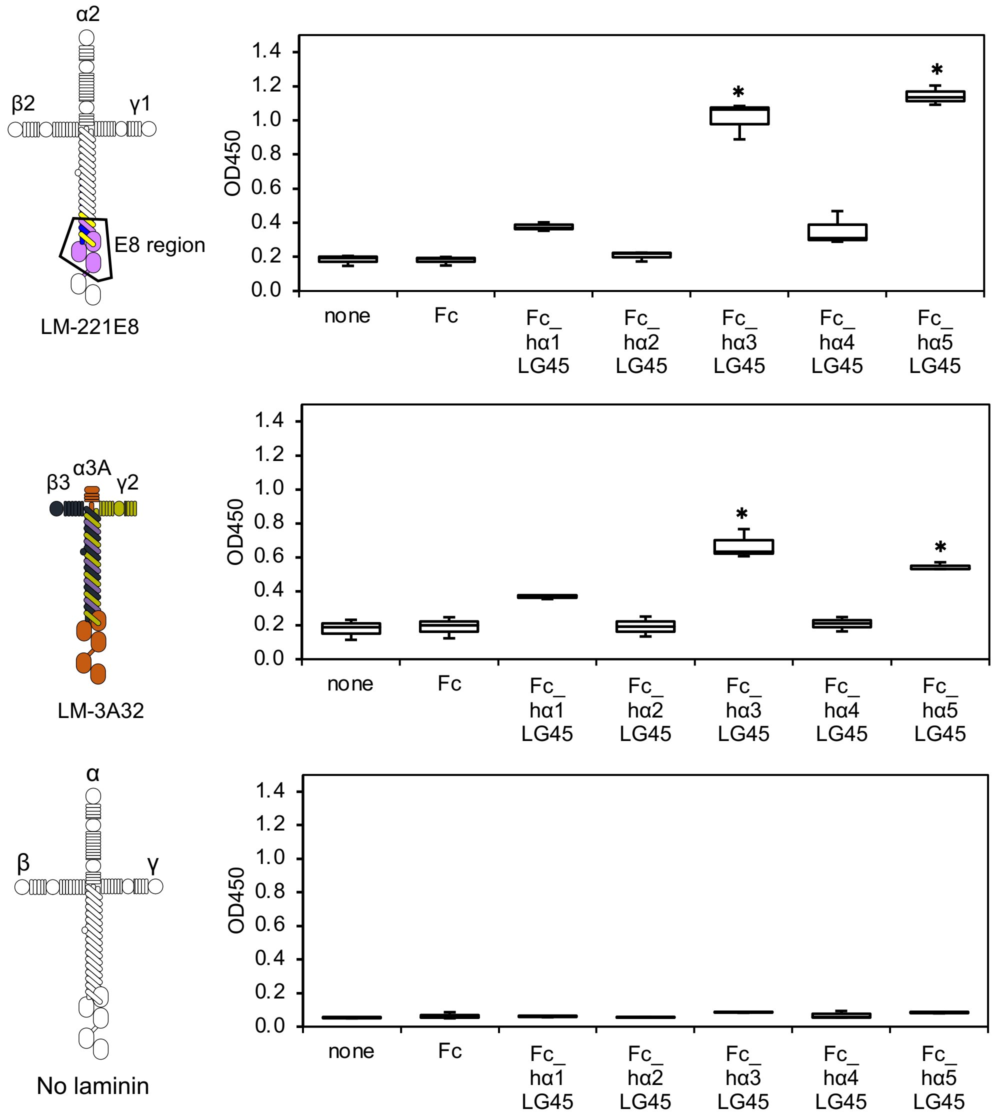

Figure S6. Solid-phase binding assays of laminin LG45 modules to the immobilized laminins. The bound recombinant proteins were detected and analyzed as described in the Methods. *$P < 0.01$.
